# Co-abundance analysis reveals hidden players associated with high methane yield phenotype in sheep rumen microbiome

**DOI:** 10.1038/s41598-020-61942-y

**Published:** 2020-03-19

**Authors:** Leila Ghanbari Maman, Fahimeh Palizban, Fereshteh Fallah Atanaki, Naser Elmi Ghiasi, Shohreh Ariaeenejad, Mohammad Reza Ghaffari, Ghasem Hosseini Salekdeh, Kaveh Kavousi

**Affiliations:** 1 Laboratory of Complex Biological Systems and Bioinformatics (CBB), Institute of Biochemistry and Biophysics (IBB), University of Tehran, Tehran, Iran; 2 Department of Systems and Synthetic Biology, Agricultural Biotechnology Research Institute of Iran (ABRII), Agricultural Research Education and Extension Organization (AREEO), Karaj, Iran

**Keywords:** Classification and taxonomy, Computational models, Metagenomics, Bacterial genes

## Abstract

Rumen microbial environment hosts a variety of microorganisms that interact with each other to carry out the feed digestion and generation of several by-products especially methane, which plays an essential role in global warming as a greenhouse gas. However, due to its multi-factorial nature, the exact cause of methane production in the rumen has not yet been fully determined. The current study is an attempt to use system modeling to analyze the relationship between interacting components of rumen microbiome and its role in methane production. Metagenomic data of sheep rumen, with equal numbers of high methane yield (HMY) and low methane yield (LMY) samples, were used. As a well-known approach for the systematic comparative study of complex traits, the co-abundance networks were constructed in both operational taxonomic unit (OTU) and gene levels. A gene-catalog of 1,444 different rumen microbial strains was developed as a reference to measure gene abundances. The results from both types of co-abundance networks showed that methanogens, which are the main ruminal source for methanogenesis, need other microbial species to accomplish the task of methane production through producing the main precursor molecules like H_2_ and acetate for methanogenesis pathway as their byproducts. KEGG Orthology(KO) analysis of the current study shows that the metabolism and growth rate of methanogens will be increased due to the higher rate of the metabolism and carbohydrate/fiber digestion pathways in the hidden elements. This finding proposes that any ruminant methane yield alteration strategy should consider complex interactions of rumen microbiome components as one tightly integrated unit rather than several separate parts.

## Introduction

The study of the complex microbial biochemical process for anaerobic production of methane and its yield is important in two opposite folds. On the one hand, the higher yield of methane is a major factor in biofuel production of methane in gas bioreactors. Many studies have focused on the improvement and stabilization of methane yield using metagenomic data.

On the other hand, methane has a great effect on global warming as it is 28 times more influential than carbon dioxide in terms of its contribution to global warming^1^ Deciphering the details of its production mechanism to propose new procedures to decline methane emission is, therefore, a topic of growing international prominence.

Grazing animals in all around the world emit a massive amount of polluting gases, including lots of methane, and domesticated ruminants, such as cattle, sheep, and goats contribute in producing the major part of these gases as approximately 86 million metric tonnes (Tg) of methane are emitted by them per year. How to encounter an increasing atmospheric concentration of methane, depends on our knowledge about its production mechanisms particularly in ruminants^2^. Also, applying any methane production mitigating procedures like different dietary formulations, feed additives, chemogenomics and, anti-methane vaccines^3^ is entirely dependent on understanding the methane production procedure^4^. Ruminants such as cows, sheep, goats, and several other animals harbor a diverse microbial community (bacteria, protozoa, fungi, and viruses) in their rumen. The members of this microbial population particularly methanogens are major players in methane emission^3^. The emergence of new technologies such as culture-independent methods like metagenomics pipelines^5^, helped scientists to unravel variations among rumen microbial communities and identify their functions.

Several metagenomic studies have been carried out to investigate methane production in different types of livestock animals. In a study by Kamke *et al*., rumen metagenome and metatranscriptome of sheep with different levels of methane emission were analyzed. Lactate-producing Sharpea spp were enriched in low methane yield sheep bacterial communities^6^ According to Wallace *et al*., the abundance of archaea genes in rumen digesta is strongly correlated with methane emissions from each animal^7^. They selected some beef cattle with high and low methane emission phenotypes and applied 16s rRNA qPCR (16S ribosomal RNA quantitative polymerase chain reaction) analysis on their samples. They showed the difference(s) between metabolic pathways of highly methane emitter’s microbiome through KO analysis. For instance, they found that archaeal genes which can influence methane production pathway (directly or indirectly) were 2.7-fold more abundant in high methane-producing phenotype versus low emitters. Another comprehensive study by Zhang *et al*. was conducted to compare the metagenome and metatranscriptome of Tibetan ruminants such as Yak and high altitude sheep, alongside with low altitude ruminants such as cattle and sheep. The research was done to find the fundamental differences of methane-producing potency of these four different types of ruminants. They observed enrichment of volatile fatty acids yielding pathways and also depression of methane yielding pathways in the rumen of high altitude ruminants. These observations might be due to the convergent evolution of rumen microbiome in order to adapt itself to different conditions^8^.

In recent years, systems biology approaches that utilize a holistic view such as gene and microorganisms networks have been using much more frequently. Accordingly, the network-based systems biology perspective could be useful in uncovering the key relationships between microbiota and different environmental factors.

Several studies have used co-expression or co-abundance networks to identify different types of taxonomic and functional correlations in various microbial environments such as soil, human gut, oceans, and rumen. For instance, Tapio *et al*. evaluated several factors such as the abundance, diversity, and co-occurrence of the ruminal microbiota in response to changes of dietary formulation in dairy cows. They created a taxonomic co-occurrence network and the results of the study indicated that no specific microbial group played a more significant role in network formation^9^. As seen, network-based approaches added new resolutions to their understanding of the diet effect on rumen microbial community and its interactions. In another study, high and low methane yield metagenomic samples of cows were taken to construct the KO co-abundance networks^4^. However, they focused on co-abundance network topological measures such as a variety of centrality measures including degree, betweenness, Bonacich and page rank centralities. Based on these measures, they found some more significant or essential genes associated with methane production in their samples. For example, they found that nodes with greater Bonacich and betweenness centralities played an essential role in methane production. However, they constructed a co-abundance network to study methane production mechanisms from a systemic perspective but they mainly emphasized single nodes with remarkable impact. It is now clear that a phenomenon such as methane production is a complex and multifactorial trait. Hence, in order to elucidate this complex mechanism, using systemic and holistic approaches is not only valuable but also indispensable. This point of view is being employed in many complex traits such as human diseases.

In general, the goal of recent studies was to find the most associated modules within the network to the subject of the study. For instance, Praveen *et al*. investigated the role of breastfeeding in the infant immune system. In this experiment, gut metagenomic data was analyzed. Using network-based systems biology approach helped them reveal key relationships between microbiota and host immunity and metabolic activities^10^. The idea of the co-abundance network has also been applied to taxonomic and functional levels. For example, Li *et al*. investigated a subject of tremendous quantities of microbial genes in the gut microbiome of type 2 diabetes patients, by mapping microbial genes to functional units. As a result of this study, novel biological comprehension into the association of gut microbiome with type 2 diabetes phenotype, were detected^11^.

Using co-abundance networks with systemic analysis can, nevertheless, result in some discoveries which cannot be achieved without holistic approaches. Reviewing previous studies demonstrates the high complexity of microbial environments and communities and our inevitable need for systemic approaches to properly analyze them. As an illustration, in a multifactorial and complex trait such as methane production, it is not reasonable to associate only some single and detached microorganisms or genes to methane production. In other words, high methane production can be regarded as a result of the complex interaction between microorganisms and genes which activates both methane production pathway and pathways which form substrates for methane production pathway. In the research by Jia *et al*., it has been reported that distinct microbial communities in methanogenic enrichment cultures can carry out the same function and lead to the same metabolic phenotypes particularly in bioconversion of lignocellulosic substrates to methane^12^. Hence, in order to cover the deficiencies of previous studies regarding methane production mechanisms in ruminants, a systemic view was applied in this study.

**Figure 1:**
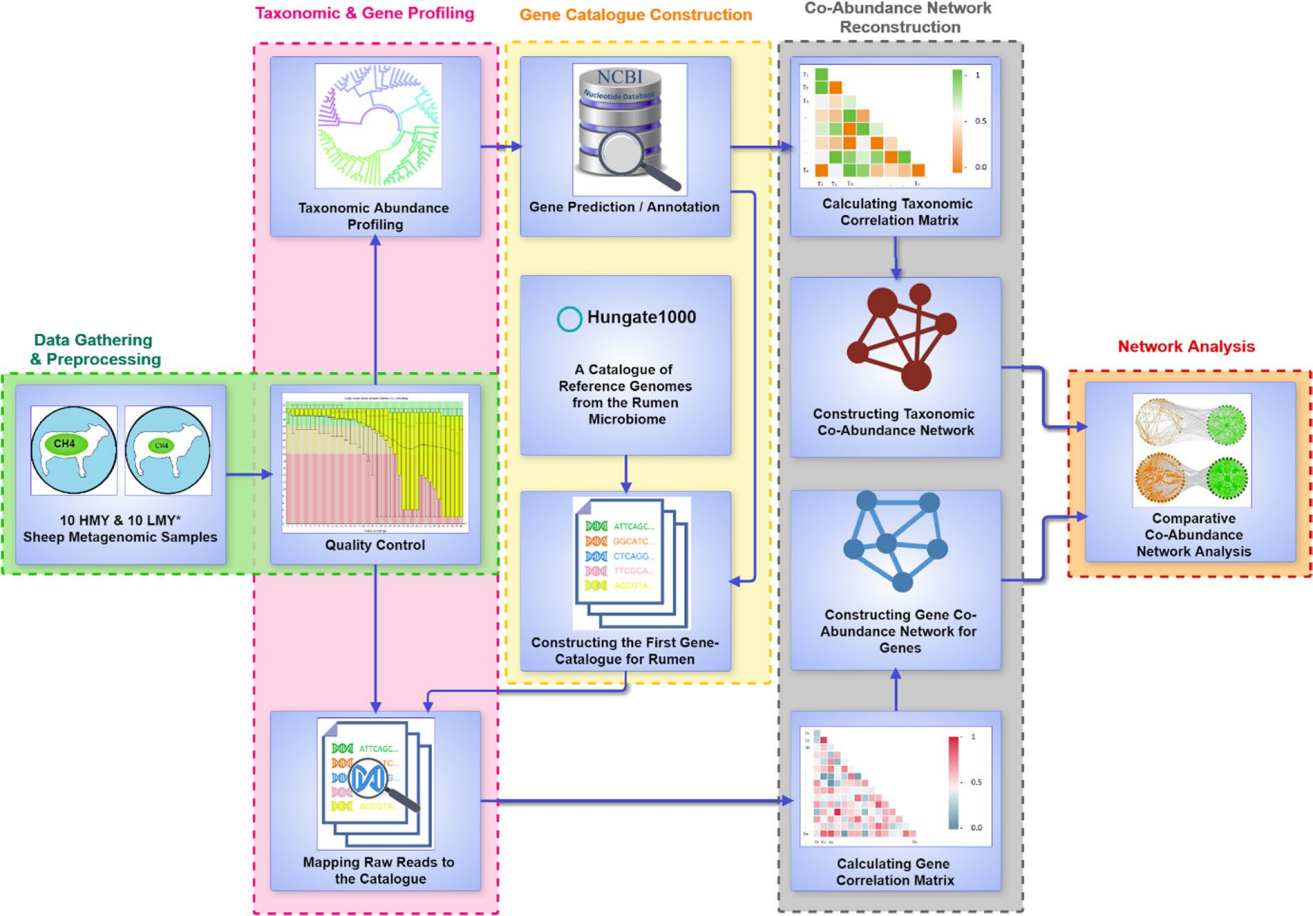
Schematic overview of constructing OTU and gene resolution co-abundance network with LMY and HMY metagenomic data.

The main idea behind this study was to investigate whether complex traits such as methane yield can be explained more precisely by simultaneously used systemic views in different layers; i.e., whether different layers of study such as OTU, gene, and KO level co-abundance networks can provide complementary insights. Thus, co-abundance networks of metagenomic samples of LMY and HMY sheep were constructed at both taxonomic and functional resolutions. Next, systemic and network-based analysis methods were utilized to decipher the modules or sub-networks associated with high methane production traits. For this purpose, samples of sheep rumen from two different phenotypes including LMY and HMY were used to identify their microbial communities as well as the most important gene clusters associated with high methane production and to detect robust associations between microorganisms and their genes within each phenotype^13^. Also, in order to construct a functional co-abundance network of rumen microbiome, we needed a comprehensive gene catalog of rumen microbiome of ruminants. To this end, we enriched the most complete available gene catalog (Hungate1000) with the metagenomics data of sheep rumen microbiome of our samples. To the best of our knowledge, this gene catalog is the most updated gene catalog for the rumen of ruminants. Although this catalog was not our main result, it can be used to identify and quantify genes of metagenomic samples from the rumen. The complete workflow of our methodological approach is illustrated in Fig. 1.

## Results and discussion

**Figure 2:**
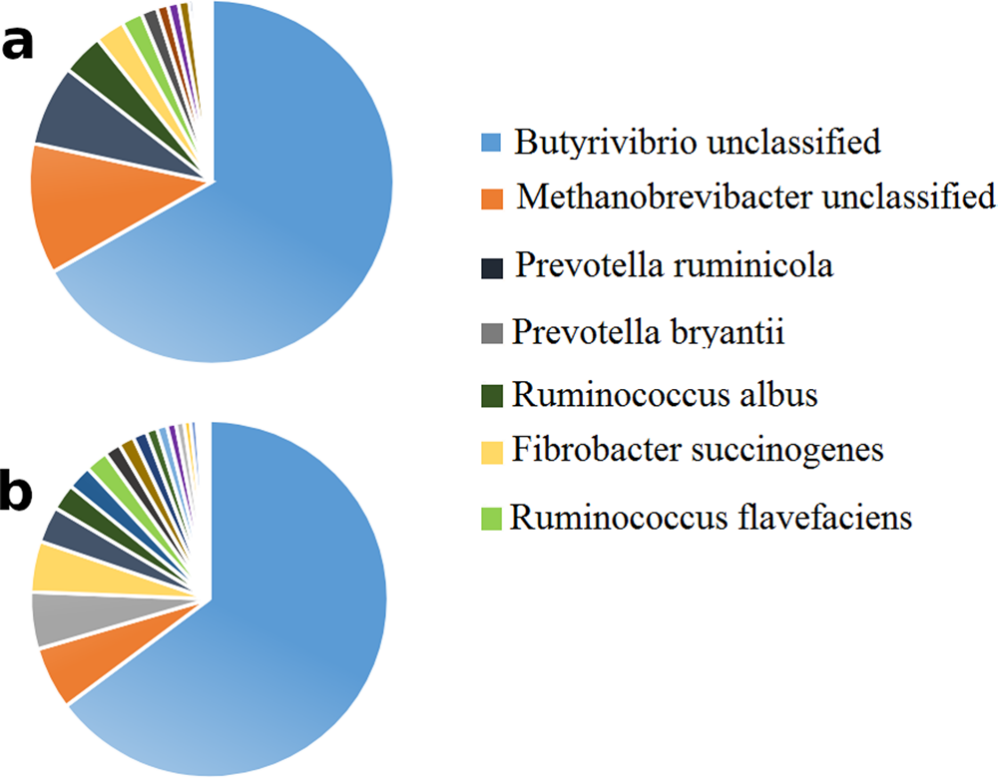
(**a**) Microbial distribution in HMY samples, (**b**): Microbial distribution in LMY samples. The legend includes only top six highly abundant bacterial species of both LMY and HMY samples.

According to the obtained results from the taxonomic binning of all 20 samples (10 samples from LMY and 10 samples of HMY phenotype), each sample contains approximately 30 unique species and the union of all uniquely identified species among 20 samples resulted in 91 microbial species. Microbial species distribution for each phenotype was elucidated in Fig. 2 as pie-charts. (Supplementary I). Based on Fig. 2, the most abundant microorganisms in the two phenotypes were almost the same. As it has been declared in the study by Hendersot *et al*., the same bacteria and archaea species dominated in almost all samples from all around the world^14^. Therefore, the mentioned result was a piece of strong evidence for the importance of having the systemic attitude for studying microbial environments such as rumen.

### Differentially abundant microorganisms between LMY and HMY samples

In this study, some species have a statistically significant difference in their abundance across LMY and HMY samples, which can be probably used as markers to separate LMY and HMY samples. Table 1 lists the differentially abundant species.

**Table 1:** Differentially abundant species among two groups (High methane yield vs. low methane yield).

Unique Species	LogFC (High Methane/Low Methane)	p-value
*Methanobrevibacter smithii*	1.57	0.0001
*Collinsella unclassified*	12.45	0.0005
*Collinsella aerofaciens*	−4.03	0.001
*Clostridium sp SY8519*	−16.94	0.001
*Desulfovibrio desulfuricans*	2.11	0.003
*Methanobrevibacter ruminantium*	1.27	0.004
*Neisseria unclassified*	−8.70	0.01
*Pseudomonas unclassified*	10.81	0.02
*Methanosphaera stadtmanae*	−1.81	0.05

### Co-abundance network analysis and module detection

#### OTU level

**Figure 3:**
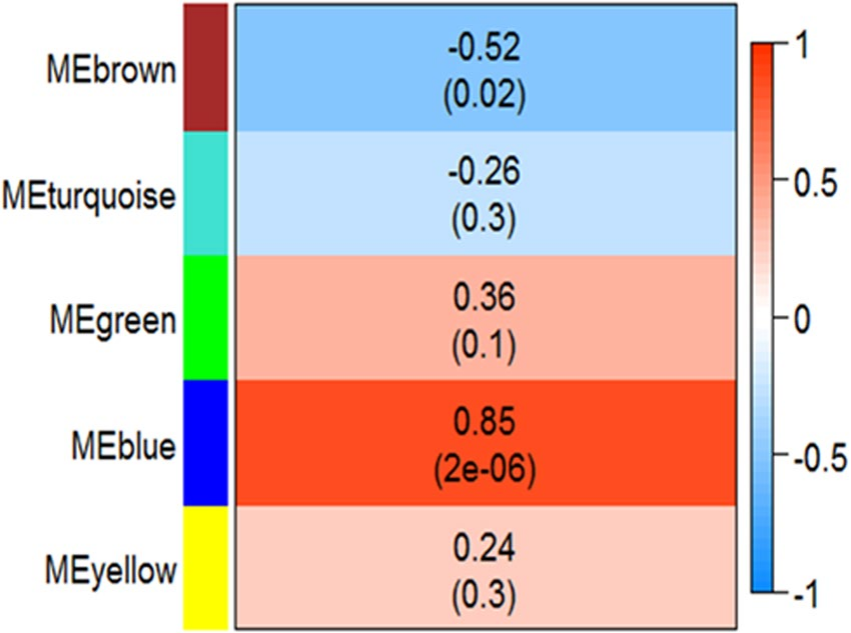
OTU level module-trait relationship. Left vertical column shows modules names, the middle column shows the correlation of each module with the trait (OTU level LMY to HMY) and the p-value of the association, also the colors in middle column are based on the correlation magnitude changing from blue for −1 to red for +1 correlation based on the right column.

At the OTU level, microorganisms were segregated into five modules (Fig. 3). Amongst, only blue module (MEblue) was statistically significantly associated with the LMY to HMY phenotype change with correlation 0.85 and high significance level. The statistically meaningful members of this module were reported in Table 2.

**Table 2:** Members of blue module highly associated with LMY to HMY phenotype difference.

OTU	Association with Low to High methane yield shift	p-value of association
*Methanobrevibacter unclassified*	0.768872	7.45E-05
*Methanobrevibacter smithii*	0.75577	0.000116
*Collinsella unclassified*	0.772537	6.55E-05
*Clostridium sp. SY8519*	−0.72188	0.000326
*Desulfovibrio desulfuricans*	0.633938	0.002687
*Methanobrevibacter ruminantium*	0.616392	0.0038

**Figure 4:**
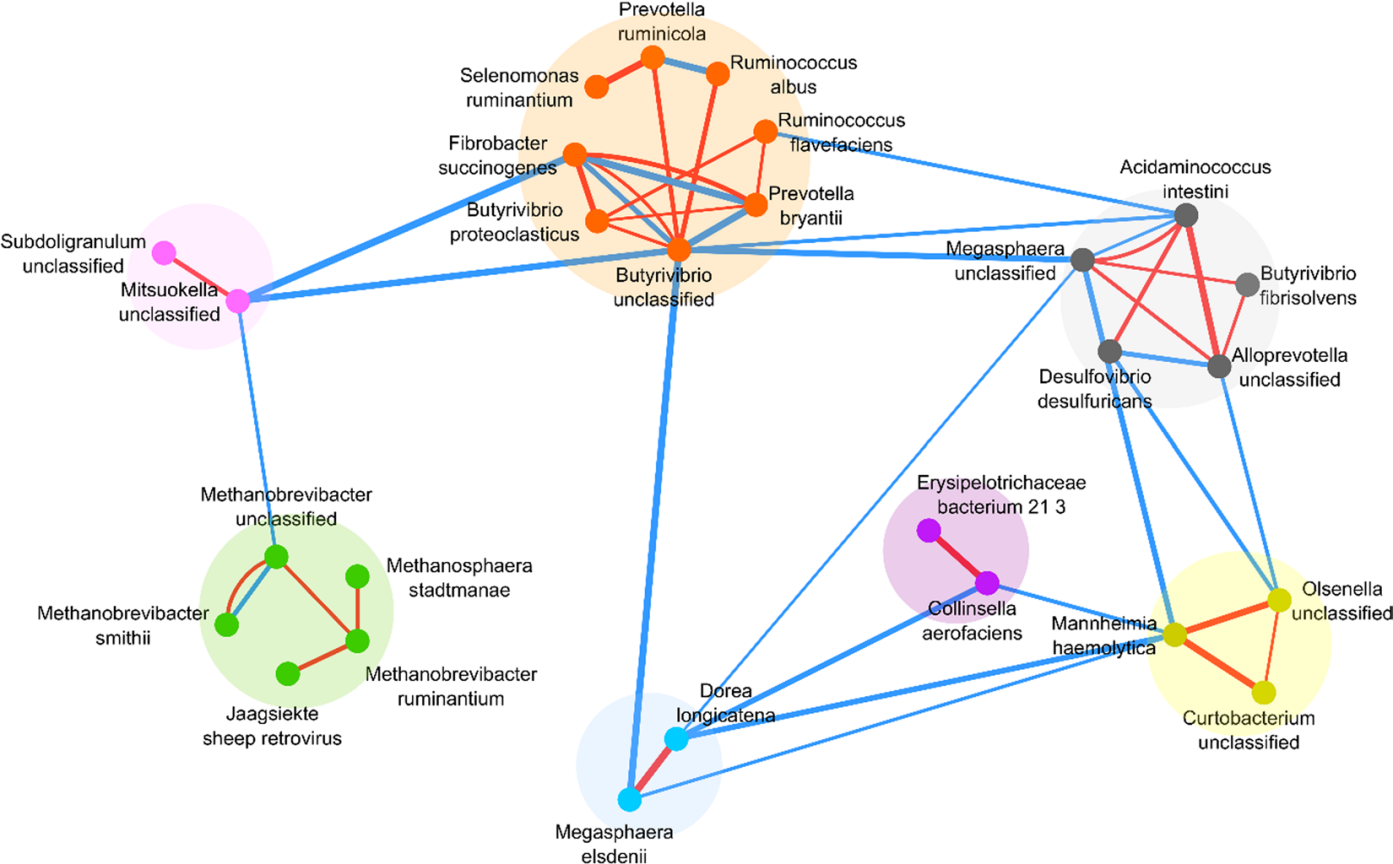
Mapping result of HMYN components on Low Methane Yield Network(LMYN) for OTU level. Each component of HMYN is colored differently. A green-colored component is a core component because of its nodes’ identity, which belongs to reported methanogens. Blue edges represent the correlations in LMYN and red edges are the connections of HMYN. The thickness of the edges represents the value of the correlation coefficient which varies from 0.70 to 0.88.

Constructing the co-abundance networks for OTUs were done with 31 species that were observed in both phenotypes. Removing the insignificant edges from the constructed network is achieved by applying a threshold to the correlation values. Keeping the edges with the mentioned threshold led to an appearance of a disconnected graph with 27 nodes, 26 edges (colored red) and seven connected components in the co-abundance network of High Methane Yield Network(HMYN) (Fig. 4).

Next, nodes of each component in OTU level High Methane Yield Network(o-HMYN) were mapped to OTU level Low Methane Yield Network(o-LMYN) and then again only the edges with a correlation equal to or more than 0.7 were kept. The result of the mapping process was shown in Fig. 4. Each component was demonstrated in different colors. The correlations between the OTUs of HMY phenotype were colored red, and the edges which were observed in the LMY phenotype were blue.

The structure of co-abundance networks for each phenotype differs significantly. Based on the network in Fig. 4, there is a strong correlation between the OTUs that belong to the same component. Four different species with the names of *Treponema bryantii, Treponema saccharophilum, Rominococcus torques*, and *Fusobacterium necrophorum* were not observed in the o-HMYN after applying the threshold.

According to mapping results, only four edges were common between 22 edges of o-LMYN and 26 edges of o-HMYN (these four edges were intra-component). The common edges showed a strong correlation between *Acidaminoccous intestini-Megasphera unclassified, Fibrobacter succinogenes-Butyrovibrio unclassified, Fibrobactersuccinogenes-Prevotella bryantii* and *Methanobrevibacter unclassified-Methanobrevibacter smithi*. Because of the existence of these four edges in both networks, it is assumed that these connections were somehow independent of the methane production phenotype. In the o-LMYN, only three edges of remained 18 edges were intra-component and specific to this phenotype and left 15 edges were all inter-component.

#### Gene level

Our constructed redundant gene catalog includes 3,972,264 genes of 1,444 different rumen microorganisms. By applying CD-HIT^15^ (parameters “-c 0.95 -aS 0.9-M 0 -T 50”) with a similarity threshold of 95% on redundant gene catalog, the number of non-redundant genes reduced to 1,777,653. Then the mentioned non-redundant rumen gene catalog was used as a reference for mapping metagenomic reads. By the end of the mapping process, 534,271 genes in LMY samples and 508,981 genes in HMY samples satisfied both mentioned criteria in the abundance profiling section. The completed tables of normalized data which gained from MOSAIK^16^ tool are available in Supplementary Table II. By obtaining the adjacency matrix of gene co-abundance network for each LMY and HMY group, 2,198 genes (nodes) were identified as the shared genes between both phenotypes. Identifying the significant edges can be done after applying a few steps of filtrations on these data to obtain a meaningful threshold.

**Figure 5:**
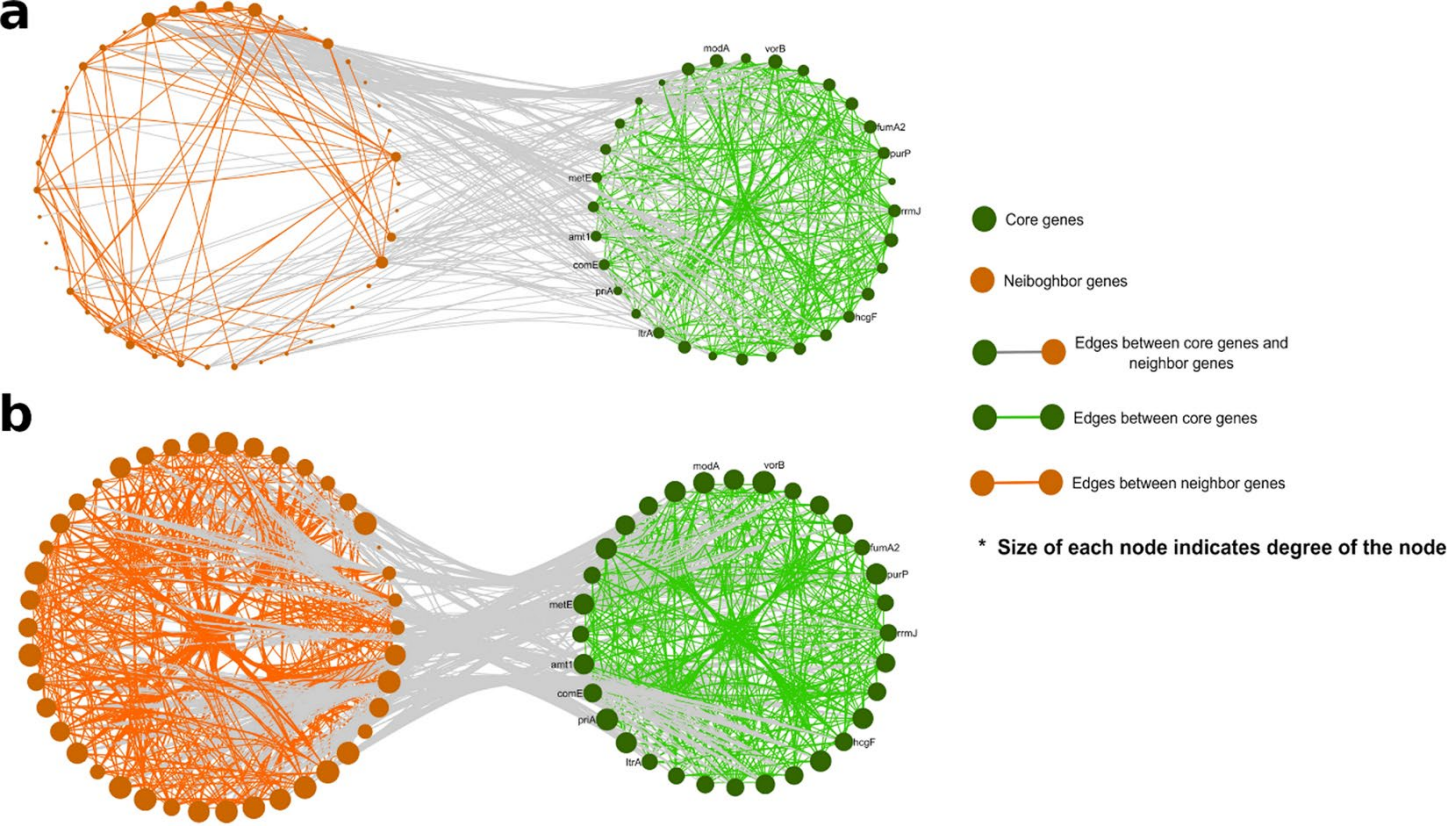
Gene resolution co-abundance network which includes only core and neighbor gene (hidden players) sets in (**a**): LMY phenotype and (**b**): HMY phenotype. The nodes without labels are predicted and newly identified genes.

After applying the 0.7 thresholds for gene co-abundance networks, 120,586 edges with 0.05 density and in Gene level Low Methane Yield Network(g-LMYN) and 154,059 edges with 0.06 density in Gene level High Methane Yield Network(g-HMYN) remained. In this state, the structures of both networks were similar to each other. To fulfill the objective of this study, the MCODE plugin^17^ of Cytoscape software^18^ was used to cluster the g-HMYN and g-LMYN networks. All modules of each network were identified and sorted based on the score which MCODE assigned to each cluster (High methane yield network Cluster1(HC1) to HC32). As mentioned before, in this study, it was assumed that the obtained information from each OTU and gene level of the co-abundance network can be complementary. For this purpose, microorganisms that have been already reported as methanogens like archaea were identified in the OTU resolution co-abundance network. All of these archaea belong to the same component with *Jaagsiekte sheep retrovirus*. In the following step, genes of these four archaea (*Methanosphaera stadtmanae, Methanobrevibacter ruminantium, Methanobrevibacter smithii*, and *Methanobrevibacter unclassified*) were identified in both LMY, HMY co-abundance gene resolution networks and counted as 65 common genes. The detected 65 genes were mapped to each g-LMYN and g-HMYN. By the end of the mapping process in HMYN, 32 genes of 65 were detected in the g-HMYN third module (HC3) and nine genes were found in the fifth module (HC5) but the remained 24 genes were scattered in the other modules. Although the first module of g-HMYN had the highest MCODE score and so was expected to contain the important genes of previously reported methanogens, none of the mentioned 65 genes were observed in high methane yield network cluster1 (HC1). HC1 only included *Butiryvibrio* genes with module density 0.9. This bacterium is a key player in the feed degradation pathways of the rumen. About half of the 65 common genes (32 genes) were detected in HC3 that all of these 32 genes belong to the same archaea species with the name of *Methanobrevibacter milleraestrain SM9*. The total number of HC3 genes was 120. There were 42 other genes in the HC3 which were neighbor nodes of previously described 32 genes and strong correlation can be detected between these two gene sets Fig. 5). In this study, 32 genes were introduced as core genes set and the mentioned 42 genes as neighbor genes set.

Densities of core genes and neighbor genes sets in HC3 of g-HMYN, were 1 and 0.878 respectively. But these values had a dramatic reduction in g-LMYN up to 0.665 for core genes and 0.114 for neighbor genes. So, in g-LMYN, the two selected gene sets did not show close and huge correlation with each other (Fig. 5).

Both of the constructed networks (OTU level and gene level) were informative on their own but considering both of them together shed more light on the complex mechanism of methane production and in other words, these two layers of the investigation were complementary for each other. Considering Fig. 3, the only highly associated OTU level module to HMY phenotype was the blue module which its members were reported in Table 2. According to Table 2, as it was expected the *Methanobrevibacter* genus was the main component of the blue module. *Methanobrevibacter* was the predominant archaea in sheep ruminant based on Table 1 and also strongly associated with the difference between HMY and LMY phenotypes. It plays a vital role in the efficient digestion of polysaccharides by consuming the final products of bacterial fermentation.

Based on our study of gene co-abundance network for LMY and HMY samples, two groups of genes with high density were discovered in the HC3 module of HMY samples. The interesting point about the core and neighbor gene was that the genes in each group were not only strongly intra-connected within each group but also there were many edges between genes of two groups.

Pondering over OTU level networks, two distinct modules with colors green and pink in Fig. 4, with a similar pattern of strong intra-connection and the same species to the gene level networks were identified. Considering both gene and OTU resolution networks can have synergistic effects. For instance, some correlations in the gene-level co-abundance network including the edges between core and neighbor gene sets cannot be inferred from the OTU level co-abundance network; although constructing a gene-level co-abundance network is costly, it is much more informative than OTU level data.

The mentioned core gene set in Fig. 5 (with green color) contained 32 genes that belonged to archaea species with the name *Methanobrevibacter milleraestrain SM9*. The second set of genes which we called neighbor genes (with orange color) in this study, contained 42 genes from different species such as *Lachnospiraceae*, *Ruminococcus*, *Butryvibrio*, and *Selemonas*. Former researches reported their function and role in the metabolism of carbohydrates and proteins repeatedly. For instance, in different studies that focus on investigating methane production mechanisms, the role of several microorganisms in protein, carbohydrates, and fatty acids digestion was examined in anaerobic reactors^19,20^.

The extracted 42 genes in the current study, can be introduced as hidden players that can have significant effects on methane production pathway. Hence, pondering on these core and neighbor gene sets, we could decode the high methane production causes with deeper understanding. We could also explain the reasons behind the co-abundance pattern in high methane yield samples. In the following, the details of our findings explained.

Microbial source of the core and neighbor gene sets were determined in supplementary table III. The significantly similar sequences to the core and neighbor gene sets were identified by using BLAST and the result has been shown in supplementary table IV. *Lachnospiraceae* and *Ruminococcaceae* which were more abundant in HMY^6^, some of their genes were presented among the neighbor genes. The role of *Ruminococcus* in sugar (starch and cellulose) digestion in the rumen has already been reported. In addition to this bacterial genus, the main functions of the other observed genes based on their microbial identity were investigated. In this regard, some bacteria such as *Butiryvibrio*, *Selenomonas*^21^, and *Clostridium* that contains 9 genes out of 42 genes in HC3 play important roles in feed digestion and metabolism because of utilizing diverse substrates such as fiber and proteins.

**Figure 6:**
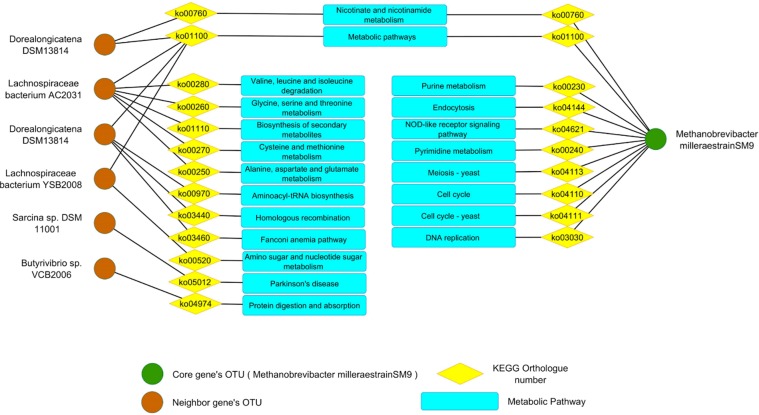
This graph illustrates the pathways that microbial species of each gene set (core gene set, neighbor gene set) is involved in based on its KOs.

Using KEGG Automatic Annotation Server (KAAS web-based tool)^22^, the KOs for both gene sets (core and neighbor) were extracted and illustrated in Fig. 6. By extracting the KO of each selected gene set, their functions and pathways that they are involved in were identified (Fig. 6).

According to the KO list, the core gene set was involved in cell cycle and DNA replication which can result in an increased number of these OTUs due to the increased rate of cellular replication in the rumen environment. KO list of neighbor gene set showed an increased rate of amino acid metabolism and protein/sugar digestion. The more the rate of sugar and protein metabolism, the more fermentation pathways will be activated which can lead to more production of fatty acids and also *H*_2_. Different types of ruminal microbial species can uptake this surplus *H*_2_ for their own needs. Among these microbial species, methanogens are the main acceptors of *H*_2_. So, after uptaking this molecule based on the below reaction by hydrogenotrophic methanogens, methane can be produced:

$$C{O}_{2}+4{H}_{2}\to C{H}_{4}+2{H}_{2}O$$

By increasing the fermentation rate of different polymers by the mentioned neighbor gene set in the rumen environment, the amount of produced fatty acids, especially acetic acid, will increase. Acetate as one the mentioned products is also another molecule which can be used by a few methanogen species in order to produce methane by Acetoclastic methanogenesis reaction as the following:

$$2C{O}_{2}+4{H}_{2}\to C{H}_{3}COOH+2{H}_{2}O$$

$$C{H}_{3}COOH\to C{H}_{4}+C{O}_{2}$$

So, this process can be another probable reason for intensive methane production in the HMY phenotype.

### Implications

Rumen plays a very important role in methane production. As a result, several kinds of research have been made to reduce the amount of methane produced by ruminants. Ruminants produce different amounts of methane which is influenced by ruminant species, the host genomic and its rumen metagenomic content and the intra and inter-connections between these two parts. As it is apparent, ruminants are controversially accepted due to their positive role in food security and negative role in climate change. Methane as a major greenhouse gas, is produced in a huge amount in the rumen and the presence and abundance of ruminal methanogenic archaea have an important effect on its production. Accordingly, proposing new strategies to select or manipulate rumen microbiome may be a proper solution for diminishing methane production. On the other hand, it is indirectly linked to the microorganisms which produce the initiators of the methane production pathway or consumes the generated methane.

There have been a lot of efforts to reduce methane emission from the rumen. Each one of the mentioned jobs tries to achieve this goal by separate approaches such as diet changes, usage of probiotics, fecal transplants and many others that mostly target the methanogenic archaea. In spite of all the previously done studies, a well-established approach for methane mitigation has not been introduced yet. Then, by developing new approaches with more focus on the microbial communities as a unit and target the pre and post methane production pathway, more reliable methane mitigation strategies will be applied. As mentioned earlier, the diet has a direct influence on the rumen microbial environment and so methane production. For example, reducing the fiber content of feed will decrease the production of methane^1,23,24^. As we mentioned in the results, the microorganisms which play essential roles in fiber digestion such as *Lachnospiraceae*, *Ruminococcus*, *Butryvibrio*, and *Selemonas*, were more frequent in HMY and also had strong interactions with themselves as well as methanogens. Accordingly, by targeting these sources, the amount of final generated methane can be diminished. Another strategy to reduce ruminal methane production is related to the usage of unsaturated fatty acids as additives to the animal diet^25,26^. In fact, the hydrogen is used by some microorganisms in the rumen in order to hydrogenate the double bonds of unsaturated fatty acids. Hence, the amount of available hydrogen will reduce and so CH_4_ production is diminished in the rumen. We can say by adding the adequate amount of fat to the diet, the community of rumen microbiome will be changed toward the reduction of fibrolytic bacteria and improvement in fat digestion which ultimately will mitigate the produced methane. As a result of applying fat to the animal, various biological pathways will be diminished. Among them, rumen fermentation, feed digestibility, and thus the methanogenic pathway that the proposed hidden players in this study are responsible for that, as well as by the direct inhibitory impact of unsaturated fatty acids hydrogenation on methanogens will be decreased which may lead to a reduction in methane production.

Most of the microorganisms that are categorized as hidden players in this study based on network and computational analyses have been already introduced as major elements of acetic acid production mechanisms in the rumen which may lead to increased methane production by previously mentioned pathways. In this regard, by interfering in the function of the proposed neighbor community function we will be able to reduce the acetic to the propionic acid ratio in the rumen and this strategy may facilitate the mitigation of ruminal methane production. There are several molecules that promote ruminal propionic acid formation by hydrogen reduction such as malate, fumarate. These mentioned organic acids are the potential feed additives for mitigating CH_4_ emission from ruminants^27–30^. The usage of some dietary probiotics that increase propionic acid production can indirectly influence the production of methane.

According to a recently published study, three different types of the ruminal microbiome in sheep have been introduced as ‘ruminotypes’. These ruminotypes showed the association with methane production changes^31^ that the ruminotype H which produce a high amount of methane includes *Ruminococcus*, other *Ruminococcaceae, Lachnospiraceae, Catabacteriaceae, Coprococcus*, other *Clostridiales, Prevotella, other Bacteroidales*, and *Alphaproteobacteria* as highly abundant microorganisms. These microbial species are also represented as a neighbor module in our study. Hence, it seems targeted interventions on the microbial networks will be a powerful method for engineering available microbial communities or creating synthetic communities in the future.

In the area of ruminal methane production, several studies have been carried out. For example, in a study by Tapio *et al*.^32^, the role of the ciliated protozoa in methane production was investigated. Removal of ciliated protozoa which produces abundant H_2_ in the rumen, results in an average 11% lower methane emissions *in vivo*. Based on their results, the abundance of archaea did not have a strong correlation with methane yield from each animal. The overall combination of the archaeal and other microbial communities and their interactions appear to have a stronger effect. Along with Tapio’s research, we investigated particularly with computational attitude, other hidden microbial communities that have a significant impact on methane production indirectly. So, here we tried to identify the key influential factors of the rumen microbiome environment leading to high or low methane emissions regardless of focusing only on methanogens because controlling the hydrogen supply to the methanogens will lead to reduced methane emissions and allow us to avert the hydrogen towards other pathways that have no role in methane production.

It is important to note that the rumen microbiome composition has been shown to have a substantial impact on the health condition and productivity of the animal along with the host genetic features. Thus, we can say the genomic and metagenomic content of ruminants and the interactions between them will ultimately form the animal’s phenotype and characteristics. Accordingly, any intervention and manipulation in the microbial community of the rumen will lead to significant consequences. Thus, this requires comprehensive and multi-dimensional researches to validate the proposed interventional approaches. In this study, we tried to deeply investigate the rumen microbial communities and their interactions from the computational and network perspective. So, the obtained results may facilitate the development of methane mitigating strategies and help other experimental researchers to narrow their research area and reduce cost. It is important to note that this study was done with 20 sheep rumen metagenome and for any global accurate results, several extensive studies that consider different types of factors and variables must be done. For example, dietary interventions in goat kids (from birth to 3-month-old) have been shown to remodel the microbial combination and influence the host phenotypes (methane emission, volatile fatty acids production) in the post-weaning period^33^.

## Conclusions

In this study, we attempted to show the significant interactions and to find the correlation in microbial species as well as their genes to understand the functions and pathways that can result in high methane yield in ruminants, particularly sheep. Based on the analysis, it might be claimed that methanogens (*Methanobrevibacter milleraestrain SM9* in this study) cannot produce an excessive amount of methane by themselves because they need the essential initiator molecules such as *H*_2_ which can be a by-product of other reactions like protein/sugar fermentation pathways. So, it is evident that OTUs or their functional elements (genes) interact with each other to fulfill the same objective as producing methane. The observed high-density values of core and neighbor gene sets in HMYN along with the strong correlation between these two sets and on the contrary low density of mentioned gene sets in LMYN can verify the vitality of considering network-based perspective when trying to determine any pathway of communities like rumen microbial environment.

Due to the fact that a large portion of microbial content and a variety of ruminal microorganisms are unknown, the investigation of the relationships between OTUs cannot precisely mirror the interactions among all components of the microbial environment. Likewise, most phenotypes associated with microbial environments are much more complex in their nature that can be deciphered only by studying the microbial abundance. Since genes are functional units of microbial environments, and many of these genes are common among different microorganisms, so the co-abundance between a subset of microorganisms does not necessarily result in the co-abundance of their genes and vice versa. In fact, systemic studies, such as the comparative study of networks at various resolutions (OTU, gene, etc.), provide a synergistic approach toward a more comprehensive understanding of complex microbial environments.

In order to carry out such studies, the preparation of a gene catalog from the ruminal environment is a major and inevitable step. In this regard, a gene catalog was created for the first time using information from the Hungate1000 project along with genetic data of other ruminant microorganisms identified in sheep rumen. The prepared gene catalog has the potential to be used as a reference to calculate the abundance of genes found in a variety of rumen metagenome samples.

In conclusion, it can be stated that for any study on the high methane yield associated phenotype of each sheep, several important factors along with their systemic interactions should be investigated. The abundance and the correlation of microorganisms and genes that directly or indirectly play a role in methane production are among the important factors to be considered in any microbiome study.

As mentioned earlier, because of the crucial importance of the rumen microbiome on the ruminant’s growth and production, any proposed methane mitigating strategy must be verified under several conditions and applied with strict caution. Because, it may lead to several unwilling consequences which may affect the animal and also the environment such as acidosis, reduction of feed efficiency in beef and dairy ruminants, massive methane production, and animal death.

## Materials and methods

Since methane production can be affected by several factors like diet, host genetics, hereditary factors, gender, age, and environment^34^ samples should only be used to study the microbiome effect on methane level emission. 20 metagenomic samples of LMY sheep and HMY sheep^13^ were downloaded from NCBI under project no. PRJNA202380. Each phenotype contained 10 samples. The data belonged to LYM and HYM sheep which were obtained at two different time intervals with 30 days’ interval. In the following step, metagenomic data was pre-processed using SRAtoolkit^35^, FastQC^36^, and BBDuk (a member of BBtools package). The MetaPhlAn2 tool was used to taxonomic profiling of metagenomics samples^37^.

### Rumen gene catalog construction

To establish a non-redundant rumen gene catalog, 442 culturable rumen microorganisms identified genes were downloaded from https://genome.jgi.doe.gov/TheHunmicrobiom ^38^. To improve our rumen gene catalog, we used MetaPhlAn2 tool to identify rumen microorganisms of LYM and HYM sheep metagenomic samples. In addition, QIIME2^39^ was applied on 45 different DNA amplicon sequenced files^13^ to detect additional new microorganisms based on their 16s rRNA genes. To predict genes of 125 microbial genus and species, first, their genomic data were downloaded from the Refseq database and then GeneMarkS (a member of MetaGene tool)^40^ was used to extract the open reading frames. At the final step of making a gene catalog, it was essential to construct a non-redundant version. CD-HIT tool with the similarity threshold of 95% was, therefore, used to remove redundant genes.

### Abundance profiling

#### OTU level

We assessed Taxonomic relative abundances of all 20 samples (10 samples from LMY phenotype and 10 samples of HMY phenotype) by MetaPhlAn2 tool (Supplementary V). Based on the taxonomic analysis using MetaPhlAn2, 91 species were identified as unique microbial species in the samples. On the other hand, some species were not determined in some samples or they were present just because of sequencing or MetaPhlAn2 errors as false-positive counts. Hence in order to eliminate potential false-positive counts, the microbial species which were only present in at least 40 percent of the samples of both LMY and HMY groups were kept for network analysis. Consequently, the number of species reduced to 31 unique microbial species. (Supplementary VI). Moreover, differentially abundant OTUs between LMY and HMY phenotype were identified using unpaired t-test in R. Statistically significantly altered abundance levels with p-value < 0.05 and absolute logarithmic fold change >1 identified within OTUs and called differentially abundant OTUs.

#### Gene level

Sample reads were mapped to the constructed non-redundant gene catalog separately by MOSAIK. So, converting BAM file to a SAM or text file was done by SAM tools package^41^. Then, a python code with the following criteria was applied to SAM files to choose the mapped reads to each gene:

1. Both ends of a paired-end reads were aligned to the same gene.
2. One end of a read was mapped to a gene while the other end of this read was not mapped to any of the other genes.

Then, we needed a formula equation for the normalization because comparing the abundance of different genes across all samples can be done only on normalized data. So, the normalization was done based on Eq. 1:

1 $${x}_{ij}=\frac{\frac{{y}_{ij}}{{l}_{i}}}{\frac{{\sum }_{k=1}^{{g}_{j}}\frac{{y}_{kj}}{{l}_{k}}}{{s}_{j}}}$$

In this equation:

*x*_*ij*_ = The normalized abundance of gene i in sample j,

*y*_*ij*_ = Absolute abundance of gene i in sample j,

*l*_*i*_ = Length of gene i,

*g*_*j*_ = Total number of genes in sample j,

*s*_*j*_ = Total number of reads in sample j.

The total number of genes in LMY and HMY gene sets includes 319,253 genes, among them almost 25,000 genes were present only in LMY samples and around 40,700 genes were only present in HMY samples. By applying several filtrations, 480 potential biomarker genes were identified for HMY and about 1,800 genes were identified for the LMY phenotype (Supplementary VII). The resulting merged gene-level abundance data table in this stage included almost 253,000 genes which were so big not only to handle but also to analyze and getting biologically meaningful data. Hence, in order to narrow down our analysis to probably the most effective and vital genes and co-abundance gene networks, t-test analysis was used. As a result, genes that were highly statistically significantly altered between LMY and HMY with p-value < 0.001 and logarithmic fold change >1 were kept. Using this approach, the number of genes was reduced to 2,198 genes (Supplementary VIII).

### Co-abundance network reconstruction based on phenotype transition

In order to show the similarity at OTU and Gene levels, Pearson’s correlation coefficient was used as the weight of edges between the two elements. We used Weighted Gene Co-Expression Network Analysis (WGCNA) R package^42^. Next, dissimilarity matrix 1-Adjacency was used as an input for hierarchical clustering by the function flushClust in WGCNA with method = “average” and cutreeDynamic function. The similarity between the abundance profile of species i and the eigengene of a module j is calculated in Eq. 2:

2 $${K}_{ME}^{j}(i)=cor({x}_{i},M{E}^{j})$$

Where *x*_*i*_ states abundance profile of species i and *ME*^*j*^ stands for the abundance profile of the eigengene of module j. Moreover to this analysis, we reconstructed OTU level co-abundance networks for low and high methane yield samples using WGCNA package. These two networks were reconstructed to identify modules associated with high methane yield phenotype and topological differences between two networks.

Similar to the OTU level co-abundance network reconstruction, we used the WGCNA package on gene abundance level data to reconstruct gene co-abundance networks. But, since all gene-level data were significantly different between two phenotypes the resulted gene modules were almost equally highly associated with LMY to HMY phenotype transition. Furthermore, we reconstructed the LMY and the HMY co-abundance network of our LMY and HMY samples respectively. These two networks were used for studying topological features between two phenotypes and more functional level analysis.

### Co-abundance network clustering in each phenotype

In order to identify the most influential modules associated with HMY and LMY phenotypes in both OTU and gene-level co-abundance network Cytoscape 3.6.0 software was used. The constructed networks by WGCNA package in both OTU and gene-level were imported to Cytoscape to further analysis. Finally, in order to find the systemic players associated with high methane yield clustering of the gene level networks must be carried out. For this purpose, the MCODE plugin of Cytoscape was used to clustering gene-level high methane yield network (g-HMYN) and gene-level low methane yield network (g-LMYN). For KO analysis, KAAS tool was used in order to find the metabolic pathways in which any genes of g-HMYN and g-LMYN co-abundance networks are contributing.

## Supplementary information

Dataset 8.

Dataset 7.

Dataset 6.

Dataset 5.

Dataset 4.

Dataset 3.

Dataset 2.

Dataset 1.

## Data Availability

The metagenomic, metatranscriptomic and 16s rRNA sequences have been downloaded from the NCBI Sequence Read Archive under the BioProject accession number PRJNA202380.
